# Mr. Chamberlaine, on Acoroides

**Published:** 1801-11

**Authors:** W. Chamberlaine

**Affiliations:** Clerkenwell


### To Dr. BRADLEY.

Dear Sir,

WHENEVER a medicine, not very generally in uſe, is found to poſſeſs valuable properties, it becomes the duty of every practitioner, who has regard to the public welfare, to make known the reſult of his experiments, that the benefits reſulting from them may be extended as far as poſſible.

Mr. Kite, of Graveſend, has favoured the world with a very circumſtantial account of the medicinal effects of the reſin of the Acoroides reſinifera, (the yellow gum of Botany Bay) which is detailed in the fourth volume of the Memoirs of the Medical Society of London, Art. IV. p. 24.

Having, at the time the work was in the preſs, the care of correcting the ſheets, in my official capacity as Secretary to the Medical Society, which I then held, I had an opportunity of ſeeing the work long before it was made public; and juſt about the time that part of the volume was printing off,* a very diſtreſſing cholera morbus, ſimilar to the preſent epidemic complaint in the bowels, was prevalent. I made trial of the Botany Bay gum; in the very firſt inſtance I found it ſo ſucceſsful, that I immediately purchaſed four pounds of it, at

* July and Auguſt 1793.

Hopkins, Jackſon, and Manley's, Pater-Noſter Row, expecting it would conſiderably riſe in price, as ſoon as the publication of the volume ſhould make it known to the world.

I have been in the habit of diſpenſing it very liberally ever ſince in autumnal complaints of the inteſtines, and indeed in general, where I have found obſtinate diarrhœa, with tendency to dyſentery, either with adults or young children, to which latter, as it has no diſagreeable taſte, it is particularly applicable.

Within theſe two months I have had above an hundred caſes of the preſent epidemic complaint of the inteſtines; and having lately, and very opportunely, received a preſent of a quantity of this yellow gum, or reſin of Botany Bay, from my much reſpected and moſt valuable friend Captain William Kent, of his Majeſty's ſhip Buffalo, lately arrived from thence, (who collected it purpoſely for me, and for which I take this public opportunity of returning him my thanks) I have been enabled to uſe it very freely, and find both the reſin itſelf, and the tincture of it, eminently to anſwer the character given of it by Mr. Kite, Dr. Bengo, Mr. Harris of Graveſend, Mr. Thompſon of Rocheſter, and other gentlemen whoſe names appear in the memoir alluded to.

I am inclined to think this gum, or reſin, would conſtitute a valuable article of commerce, independently of its excellence as a medicinal drug, for the tincture I made of it, a highly ſaturated ſolution of the purer parts in rectified ſpirit, digeſted in the ſolar heat for three months, makes a very excellent and beautiful yellow lacquer, or varniſh, that reſiſts the power of hot water, and is not eaſily ſcraped off any piece of furniture that it is laid on.

The only impediment againſt the acquiſition of any knowledge as to how many uſes this gum might be applied, would be, that as ſoon as it ſhould become known that this article is good for ſomething, a heavy duty, equal almoſt to a prohibition, would be laid on its importation.

I have the honour to be, with much reſpect,

Dear Sir,

Your very humble ſervant,

*Clerkenwell, Oct. 12, 1801.* W. CHAMBERLAINE.

---

*Copy of two Letters from William Darton, Bookseller, No. 55, Gracechurch Street.*

Friend Chamberlaine,

Harraſſed by a pain in my face for nearly a month paſt, greatly fatigued by loſs of reſt in attending my buſineſs, and quite quite debilitated by a dyfentery, caufing ten ftools in twenty-four hours, I have an inclination to try the yellow gum from Botany Bay. Pray prepare me fome, and a fmall aromatic opiate* at night, for I fleep very indifferently; and whether better or worfe by the trial, thou mayeft expect to receive my acknowledgements, and any pay required from

Thy friend,

*7 mo.* 18, 1793. W. DARTON.

P. S. I am fo faint I can hardly write.

Three days afterwards I received the following.

W. Darton having found great and fudden relief from the pills, and knowing feveral of his friends now afflicted as he was, requefts his friend Chamberlaine to fend him fome of the tincture of the gum, as mentioned in page 55 of Medical Memoirs. If that is now impracticable, a double portion of the pills, not quite fo large as the laft, will be acceptable. W. D. has quite loft the pain in the abdomen, though it had been very violent indeed; three pills relieved that, and the violence of the lax is abated.

*Gracechurch Street, 7 mo.* 23, 1793.

